# Compression of FASTQ and SAM Format Sequencing Data

**DOI:** 10.1371/journal.pone.0059190

**Published:** 2013-03-22

**Authors:** James K. Bonfield, Matthew V. Mahoney

**Affiliations:** 1 Wellcome Trust Sanger Institute, Cambridge, United Kingdom; 2 Dell Inc., Round Rock, Texas, United States of America; Thomas Jefferson University, United States of America

## Abstract

Storage and transmission of the data produced by modern DNA sequencing instruments has become a major concern, which prompted the Pistoia Alliance to pose the SequenceSqueeze contest for compression of FASTQ files. We present several compression entries from the competition, Fastqz and Samcomp/Fqzcomp, including the winning entry. These are compared against existing algorithms for both reference based compression (CRAM, Goby) and non-reference based compression (DSRC, BAM) and other recently published competition entries (Quip, SCALCE). The tools are shown to be the new Pareto frontier for FASTQ compression, offering state of the art ratios at affordable CPU costs. All programs are freely available on SourceForge. Fastqz: https://sourceforge.net/projects/fastqz/, fqzcomp: https://sourceforge.net/projects/fqzcomp/, and samcomp: https://sourceforge.net/projects/samcomp/.

## Introduction

Data volumes from next-generation sequencing instruments are a major issue for storage and data transfer costs. Between 2008 and 2012 sequencing costs dropped 1000 fold (K. A. Wetterstrand, http://www.genome.gov/sequencingcosts/ accessed on June 26, 2012) giving an approximate cost halving every 5 months. A long term trend for storage shows a cost halving every 14 months (M. Komorowski, http://www.mkomo.com/cost-per-gigabyte accessed on August 20, 2012). It is tempting to believe that data compression will resolve these problems, but with exponential growth rates it can do no more than delay the inevitable time when organizations will need to consider whether they truly need to retain everything. However, a good case can be made [1] that some samples will always be worth storing in their raw DNA sequence form. Improving sequence compression is an essential part in reducing the dependency on storage and network bandwidth.

In October 2011 the Pistoia Alliance formally announced a competition to compress next-generation sequencing data. Entries to the competition were run on a virtual machine in the Amazon Cloud (http://aws.amazon.com/) against a private test set, with results publicly displayed on a leader-board (at http://www.sequencesqueeze.org) throughout the competition. The data to be compressed was in FASTQ format [2], an industry standard format supported by a wide variety of next generation sequencing manufacturers including the data tested here, produced by Roche 454 [3], Life Technologies SOLiD [4] and Illumina GA/HiSeq [5].

There has been considerable work on compression of sequencing data, with some researchers specializing only on sequence compression [6], [7] or quality value compression [8], [9] with others supporting full FASTQ file compression; G-SQZ [10], SlimGene [11], SOLiDzipper [12], DSRC [13], Quip [14], SCALCE [15] and KungFQ [16]. Related to this is work on SAM/BAM [17] compression including Goby (F. Campagne, http://campagnelab.org/software/goby/ accessed on July 19, 2012), CRAM [18], SAMZIP [19] and NGC [20]. Overviews of compression within bioinformatics can be found in [21] and [22].

We compare our work only against other full FASTQ and SAM file compressors and against more general purpose compression algorithms like gzip [23] and bzip2 (J. Seward, http://www.bzip.org accessed on August 16, 2012). Both authors submitted multiple entries to the SequenceSqueeze competition. J. Bonfield submitted the Fqzcomp and Samcomp variants while M. Mahoney submitted Fastqz variants. Fqzcomp and Fastqz both accept FASTQ files as input, with the latter also taking an optional genome sequence to perform reference based compression. Samcomp also performs reference based compression but requires previously aligned data in the SAM format instead.

## Results

### Overview

**Figure 1:**
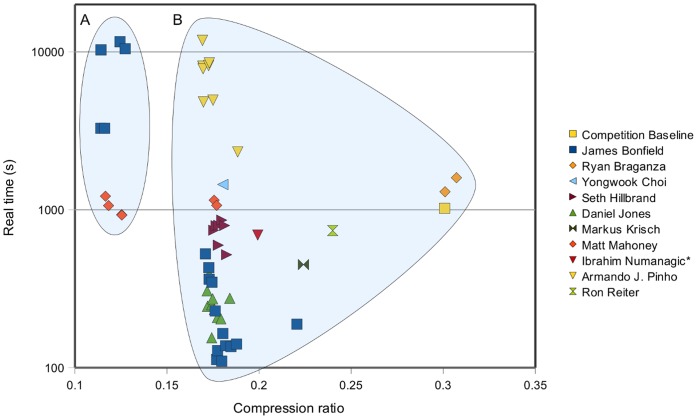
SequenceSqueeze results: real time vs compression ratio. Each mark represents a different entry, coloured by author. Ibrahim Numanagic’s entry had a minor decoding problem causing a minority of read-names to mismatch. All other entries plotted here were lossless. The entries have been broken down into reference based (A) and non-reference based (B) solutions. A clear wall can be seen in the non-reference methods requiring exponential growth in CPU time for minor linear improvements in compression ratio.

A brief summary of all successful entries to the SequenceSqueeze competition can be seen in figure 1. We observe a wall where entries were unable to improve on compression ratio without an exponential increase in both CPU time and memory. We believe this wall represents close proximity to the Kolmogorov complexity [24], beyond which further compression is only achievable via lossy methods or use of additional knowledge such as the target genome. More information on the contest results is available in the supplementary material.

We further compare and analyze the programs presented here – Fastqz, Fqzcomp and Samcomp – to the other top entrants of the SequenceSqueeze contest (Daniel Jones’ Quip and the IEETA’s SeqSqueeze1 programs), the previously published DSRC tool and the general purpose gzip and bzip2 compression programs.

With most entrants to the competition submitting multiple entries, and seeing the results live, there may have been accidental over-fitting of the (unseen) test set. Hence we tested the programs against a number of other public data sets. We chose a representative subset of the same data used in the DSRC paper along with SRR065390_1.

The data sets used for testing are presented in Table 1. Not all programs tested supported all types of data. SRR003177 consisted of variable length 454 data with some (erroneously) up to 4 Kb long. Fastqz cannot deal with variable length data while SeqSqueeze1 has a sequence length limit of 1 Kb which we increased to allow this data to compress. SRR007215_1 contains SOLiD colour-space data. This is not supported by Quip or Fastqz, while Fqzcomp needed fixes to support a fastq format variant used in this data set. SRR027520_1 is a low coverage 1000 Genomes project Illumina run [25]. SRR065390_1 is a 33 fold coverage Caenorhabditis Elegans genome, chosen to demonstrate compression ratios on smaller and deeper genomes. All programs supported the Illumina data sets.

**Table 1:** Data sets used for program evaluation.

Run ID	Platform	Species	No. Seqs	Length	File size	Depth
SRR003177	454 GS FLX Titanium	Human	1,504,571	564	1,754,042,560	0.28x
SRR007215_1	ABI SOLiD System 2.0	Human	4,711,141	25	689,319,444	0.04x
SRR027520_1	Illumina GA II	Human	24,246,685	76	5,055,253,238	0.61x
SRR065390_1	Illumina GA II	C.Elegans	33,808,546	100	8,819,496,191	33.8x

The data sets used to test the compression tools along with the sequencing platforms that produced them. *Length* is the average sequence length. *Depth* is the average genome depth assuming 100% of sequences in the data set can be aligned.

Table 2 shows the program names, versions and command line arguments used for testing. These were the latest versions at the time of manuscript preparation.

**Table 2:** Program names, versions and options.

Name	Version	Compression mode	Options
SCALCE	2.3	fast	-B 1G -T 2
		slow	-c bz -T 2
dsrc	1.01	fast	
		slow	-l -lm2048
SeqSqueeze1	1.0(svn)	slow	-h 4 1/5 -hs 5 -b 1∶3 -b 1∶7 -b 1∶11 -b 1∶15 1/20 -bg 0.9 -N -s 1∶1 -s 1∶2 1/5 -s 1∶3 1/10 -s 1∶4 1/20 -ss 10 -sg 0.95
fastqz	1.5	fast	e
		slow	c
fqzcomp	4.4	fast	-n1 -q1 -s1
		medium	-n2 -q2 -s6
		slow	-n2 -q3 -s8+ -b
quip	1.1.1	fast	
		slow	-a
sam_comp1	0.7	–	
sam_comp2	0.3	–	
cramtools	1.0	–	–preserve-read-names -L m999
goby	2.01	–	-x MessageChunkWriter:codec = hybrid-1–preserve-soft-clips –preserve-read-names –preserve-all-mapped-qualities
samtools	0.1.18	–	
gzip	1.3.12	–	
bzip2	1.05	–	

Program version names, numbers and common command line options. Additional options were sometimes required to specify the name of the reference used, but this differed per data set.

### Non-reference Based

**Figure 2:**
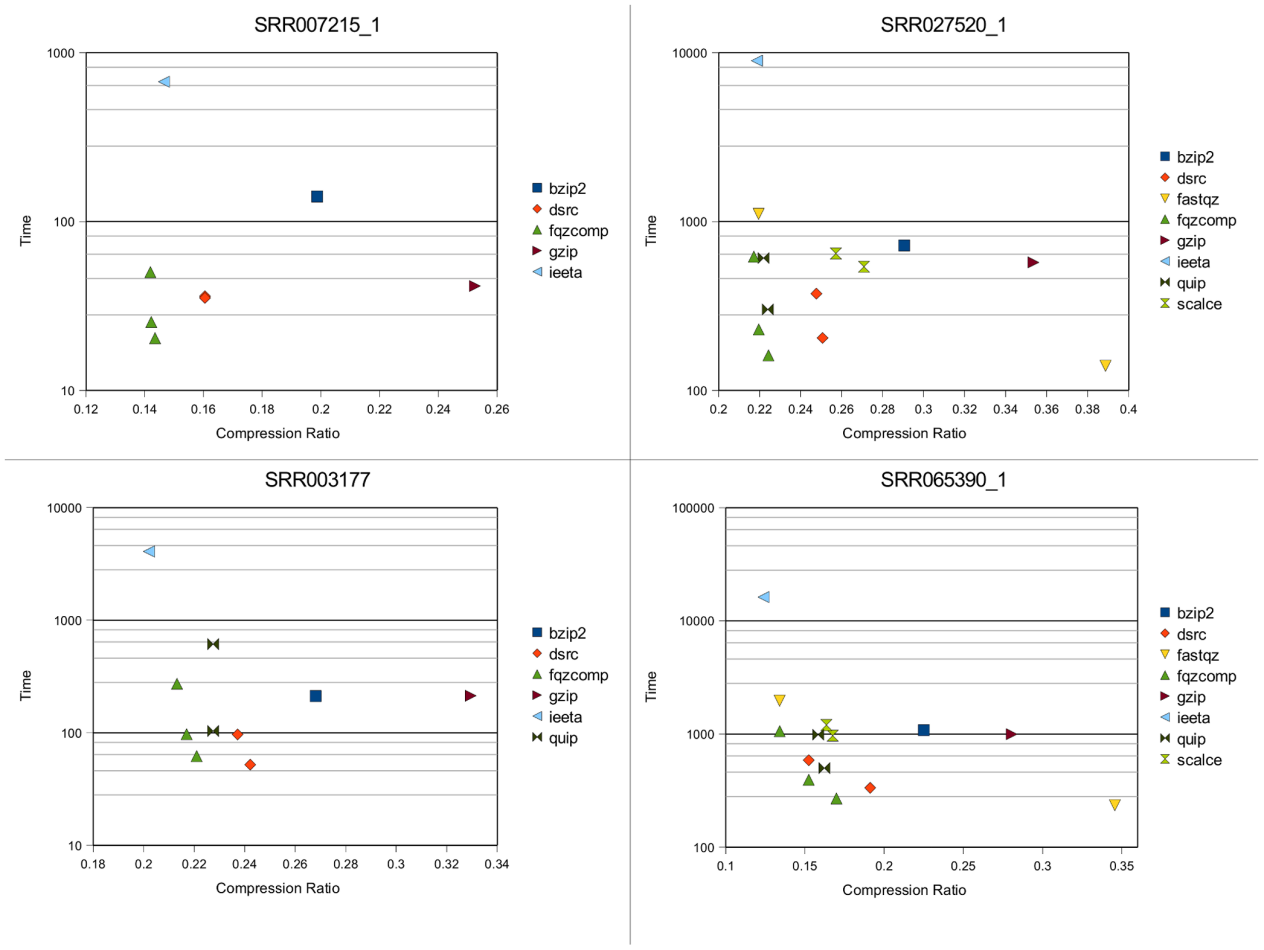
File size ratios vs real time to compress. SRR007215 is SOLiD data, SRR003177 is 454 data, while SRR02750 and SRR065390 are Illumina data at shallow and deep depths respectively. Not all programs support all types of data.

Figure 2 shows the non-reference based compression of the four data sets. Some programs are presented more than once showing the effect of adjusting the command line options. All tests were performed on a machine with Intel Dual-Core E5300 CPU (2.6 GHz) with 6 GB of memory, running the Ubuntu 10.04 Linux operating system.

Table 3 shows the same data in more detail including memory consumption and decompression times. We see a clear trade-off between time, memory and compression ratio. The fastest and lowest memory programs tend to produce larger files. Largest of all is Fastqz in fast mode. This only performs the preprocessing steps to quickly pack multiple bases and confidence values together without any subsequent encoding and compression. In isolation it is not particularly small. It may be improved by applying a fast general purpose compression program to the output files, but this option was not explored. Next largest are the general purpose gzip and bzip2 programs. With the exception of the very quick decompression in gzip these tools look like a poor trade-off between size and speed with several tools outperforming them in all metrics, demonstrating that file format specific knowledge is important for efficient compression.

**Table 3:** Compression rates and ratios.

		SRR003177 (LS454)				SRR007215_1 (SOLiD)			
Program	Mode	Ratio	C.R.	D.R.	Mem	Ratio	C.R.	D.R.	Mem
gzip		0.3295	8.2	**91.6**	**1**	0.2524	16.6	**111.6**	**1**
bzip2		0.2681	8.3	12.0	7	0.1987	4.9	23.1	7
SCALCE	fast	(a)				(b)			
	slow	(a)				(b)			
DSRC	fast	0.2422	**33.7**	51.1	61	0.1605	19.1	55.3	11
	slow	0.2372	18.2	47.1	1979	0.1605	19.5	51.7	11
quip	fast	0.2275	16.9	15.0	398	(b)			
	slow	0.2275	2.9	2.8	766	(b)			
fastqz	fast	(a)				(b)			
	slow	(a)				(b)			
fqzcomp	fast	0.2236	28.3	34.1	40	0.1455	**33.8**	54.9	**39**
	medium	0.2170	18.1	19.4	312	0.1422	27.2	38.2	310
	slow	0.2132	6.5	6.5	4407	**0.1419**	13.8	16.8	4405
SeqSqueeze1	slow	**0.2021**	0.4	0.4	4587	0.1465	1.1	1.1	4888
		**SRR027520_1 (Illumina)**				**SRR065390_1 (Illumina)**			
**Program**	**Mode**	**Ratio**	**C.R.**	**D.R.**	**Mem**	**Ratio**	**C.R.**	**D.R.**	**Mem**
gzip		0.3535	12.2	**45.4**	**1**	0.2805	8.9	**44.3**	**1**
bzip2		0.2905	7.0	13.0	7	0.2250	8.2	14.1	7
SCALCE	fast	0.2709	9.4	25.4	2212	0.1675	9.1	26.1	2181
	slow	0.2572	7.8	13.1	5162	0.1635	7.3	15.8	5257
DSRC	fast	0.2507	24.7	32.9	18	0.1912	26.4	33.2	20
	slow	0.2477	13.5	32.2	1058	0.1524	15.0	33.9	1965
quip	fast	0.2240	16.8	13.7	396	0.1622	17.7	14.5	391
	slow	0.2219	8.3	10.9	777	0.1584	8.9	11.7	775
fastqz	fast	0.3887	**36.1**	32.8	**1**	0.3456	**37.1**	30.6	**1**
	slow	0.2195	4.6	3.8	1459	0.1340	4.7	3.8	1527
fqzcomp	fast	0.2243	31.4	32.5	44	0.1733	32.7	29.4	40
	medium	0.2196	22.0	21.7	312	0.1524	22.4	20.8	311
	slow	**0.2172**	8.2	8.3	4407	0.1341	8.3	8.5	4406
SeqSqueeze1	slow	0.2187	0.6	0.6	4919	**0.1239**	0.5	0.5	4930

SRR003177 is 1.5 M human sequences of variable length (avg 564 bp); SRR07215_1 is 4.7 M human seqs of length 25 bp plus 1 primer base; SRR027520_1 is 24.2 M human seqs of length 76 bp; SRR065390_1 is 33.8 M C.Elegans seqs of length 100 bp. Ratio is the compressed size divided by the uncompressed size. C.R. and D.R. are compression and decompression rates in MB/s. (a) Program does not support variable length sequences. (b) Program does not support SOLiD data.

The fast and medium compression speeds of Fqzcomp demonstrate comparable or better speed than DSRC and SCALCE while achieving significantly higher compression ratios. However note that DSRC provides random access to the compressed files. DSRC was tested with and without the LZ encoding step. On shallow data (where most genome bases are covered 1–2 times only) it can be seen to have minimal impact on compression ratios, while harming compression speed. However LZ encoding demonstrably gives a substantial improvement on small and/or deeply sequenced genomes.

For highest compression ratio there is no clear winner with Fqzcomp, Fastqz and SeqSqueeze1 varying in rank by data set. The context mixing used in the IEETA SeqSqueeze1 entry comes at a large cost in CPU usage making it less useful. Fastqz also utilizes context mixing but the use of multi-threaded code and initial preprocessing to reduce the input data volumes offset the CPU costs considerably. Fqzcomp is the fastest of the three and comparable to the best compression ratios on all data sets except the deep C. Elegans set, but requires high memory to achieve high compression ratios.

To get a better understanding of the relative strengths and weaknesses we chose SRR027520_1 and SRR065390_1 to study the compression of individual identifier, sequence and quality components. These data sets were chosen due to being supported by all programs. We also tested these data sets using reference based compression tools. Table 4 provides a break down by data type in a variety of conditions, showing the average number of bits per complete identifier, per individual base-call and confidence value.

**Table 4:** Compression by data type.

			SRR027520_1						SRR065390_1					
Prog	Ref	Sort	Ratio	ID	Base	Qual	C.R.	Mem	Ratio	ID	Base	Qual	C.R.	Mem
Raw FASTQ	N	ID	1.0000	419.9	8	8			1.0000	454.9	8	8		
Fastqz	N	ID	0.2195	11.7	1.71	2.96	3.8	1459	**0.1340**	15.6	**1.11**	1.53	3.8	1527
Fqzcomp(medium)	N	ID	0.2196	11.3	1.72	2.95	**22.0**	**312**	0.1524	14.8	1.52	1.52	**22.4**	**311**
Fqzcomp(slow)	N	ID	**0.2172**	11.3	**1.68**	**2.94**	8.2	4407	0.1341	14.8	1.16	**1.49**	8.3	4406
Quip	N	ID	0.2219	**11.2**	1.78	2.95	8.3	777	0.1584	**14.7**	1.64	1.51	9.0	776
Fastqz	Y	ID	0.1816	**11.7**	0.88	2.96	3.2	1365	**0.1000**	**15.6**	**0.40**	1.53	4.7	1352
Samcomp2	Y	ID	**0.1810**	**19.4**	**0.75**	**2.94**	**13.6**	**1079**	0.1022	19.9	0.43	**1.49**	17.1	**365**
Quip	Y	ID	0.1885	22.2	0.90	2.95	**16.4**	1515	0.1088	21.3	0.54	1.52	**19.1**	807
Fastqz	N	pos	0.2414	52.1	1.66	2.95	3.2	1527	0.1397	64.1	0.74	1.54	4.0	1527
Samcomp1	N	pos	**0.2360**	**49.8**	**1.59**	**2.94**	**15.1**	315	**0.1147**	58.7	**0.29**	1.50	**21.8**	288
Samcomp2	N	pos	0.2628	**49.8**	2.18	**2.94**	13.5	341	0.1982	58.7	2.04	**1.49**	15.2	341
Quip	N	pos	0.2453	50.5	1.78	**2.94**	9.3	776	0.1890	**58.6**	1.83	1.53	11.2	775
SAMtools (BAM)	N	pos	0.4013	137.8	2.79	4.21	13.7	**1**	0.2344	150.9	0.94	2.47	16.7	**1**
Fastqz	Y	pos	0.2009	52.1	0.77	2.95	2.9	1406	0.1184	64.1	0.29	1.54	4.4	1352
Samcomp1	Y	pos	**0.1852**	49.8	**0.47**	**2.94**	15.7	**378**	**0.1116**	58.7	**0.23**	1.50	**21.9**	**296**
Samcomp2	Y	pos	0.1920	49.8	0.62	**2.94**	14.2	1079	0.1163	58.7	0.33	**1.49**	20.1	365
Quip	Y	pos	0.1926	**49.2**	0.64	**2.94**	**16.6**	1516	0.1165	**58.6**	0.32	1.53	19.6	808
Goby^a^	Y	pos	0.2706	99.5	0.62	4.01	4.8	1797	0.1587	110.6	0.28	1.93	6.8	1250
CRAM	Y	pos	0.2504	92.1	0.58	3.71	5.0	1514	0.1676	105.9	0.27	2.17	7.9	898

Showing the compressed file size break down by bits per sequence identifier, per base-call and per quality value. In some cases these sizes refer to cases where a reference was previously used to map, but it has not been used during compression (e.g. BAM). The ID, Base and Qual columns are the number of bits required to store the complete sequence identifier, a single base nucleotide and a single quality value respectively. The C.R. column is the compression rate in MB per second. Mem is the amount of memory required during compression. References used were human hg19 and C.Elegans WS233. Non-reference based Quip used the “-a” assembly option for high compression mode.

a Goby does not store unmapped data. The Goby figures have been estimated by adding 2 bits per absent base-call and scaling up the name and quality figures by the percentage of unmapped reads.

Fastqz outputs separate files per data type while Fqzcomp and Quip report separate figures upon completion. For other tools we either modified them to omit specific data types (Samcomp ) or produced multiple data files with identifiers, sequences and/or quality values absent and measured the difference to derive the approximate size of each component.

With the fastq in the original order as downloaded from the NCBI and without using a reference to align against, we see that Fqzcomp and Fastqz are largely comparable in size. Quip is also comparable on shallow data, but is unable to exploit the high sequence redundancy in the deep data set.

Keeping in the original name-sorted order but aligning against a reference shows some disparity. As Fqzcomp has no support for reference base compression and Samcomp1 requires genome position sorted data we only compare Fastqz, Samcomp2 and Quip. Note that Quip in the original form as submitted to the competition did not support SAM format or reference based compression. Samcomp2 and Fastqz, in this configuration of using a reference against unsorted data, came top in the SequenceSqueeze contest by compression ratio. The sequence name encoding in Samcomp2 is weaker then Fastqz, but on shallow data the sequence encoding is better, leading them to trade places between the two data sets with no overall leader. Quip is in third place on both data sets but is slightly faster. As expected in all three cases it is clear that the improvement to base-call storage is considerable, becoming the smallest component of total file size (while being the most significant and useful component).

More interesting are the results of sorting the aligned data by the position within the genome, but without utilization of the genome sequence as a reference to encode differences against. It may seem strange to not exploit this knowledge, but this use case represents storage of de-novo sequence assemblies for which no reference is known and is also the the usual form of encoding within BAM files. BAM is considerably larger for all components, particularly so with sequence identifiers. It should be noted again that these sequence identifiers are long as they contain the NCBI “SRR[*num*]” identifier (now in a random order) as well as the original machine produced identifier. Except for SAMtools all programs in this test have roughly comparable identifier and quality encoding metrics, with the significant differences coming in sequence encoding. Samcomp1 significantly outperforms the other tools, most notably so in the deep C.Elegans data set giving just 0.29 bits per base - close to the 0.23 best case when a reference is specified. This is due to the use of per-position models.

### Reference Based

Finally moving to reference based compression of position sorted files allows a direct comparison to existing reference based compression programs; CRAM, Goby and Quip. Fastqz operates on a FASTQ file and includes its own fast alignment algorithm. Some metrics on the performance of this can be seen in Table 5. All the other tools listed operate on SAM or BAM files containing previously generated alignments. The time taken to generate these alignments has not been taken into consideration in the compression rate figure.

**Table 5:** Fastqz alignment benchmarks.

Data type	Mode	Unaligned size	Aligned size
Identifier	fast	251,697,610	251,697,610
Alignment	fast	n/a	183,313,663
Sequence	fast	639,049,273	49,174,693
Quality	fast	867,178,255	867,178,255
Total	fast	1,757,925,138	1,351,364,221
Identifier	slow	47,861,283	47,861,283
Alignment	slow	n/a	105,063,319
Sequence	slow	503,239,070	30,852,888
Quality	slow	574,112,937	574,112,937
Total	slow	1,125,213,290	757,890,427

Size of data components in the public SequenceSqueeze test set SRR062634 (6,345,444,769 bytes uncompressed).

All programs perform well for base compression, although the clear winner in this category is the per-position model used in Samcomp1. As before, identifiers and quality strings are comparable between Fastqz, Samcomp and Quip, but we can see CRAM and Goby are considerably larger on quality values and, where applicable, sequence identifiers. Note however that both CRAM and Goby are random-access file formats unlike Fastqz, Samcomp and Quip. This will account for some, but not all of the size difference. Speed-wise recall that Fastqz is performing its own alignment while the other tools are operating on already aligned SAM/BAM files, making it hard to compare the overall performance.

In the context of the SequenceSqueeze competition it is clear that the long identifiers hampered file sorting. The inclusion of the NCBI identifiers added an additional 25 bits of data per identifier which could only be negated if the data was kept in the original order. This ultimately lead to Samcomp2 being the competition winner in terms of compression ratio, but it is arguably a weaker tool than the original Samcomp implementation. Generally sequencing data is either in an unsorted FASTQ file or an aligned and hence sort-able alignment file (e.g. BAM). Therefore we would not advise general use of Samcomp2. It is commendable that Quip was capable of running in all four categories listed in Table 4 and while never reaching top compression it was usually close (with deep data being the notable exception) and with acceptable speed.

It is important to note the limitations of Samcomp. While it uses SAM/BAM as input and output formats, it is not a full SAM/BAM compressor. It was designed with the SequenceSqueeze competition in mind and so primarily focuses on identifiers, sequence and quality values. SAM flags and mapping scores are also stored, but the SAM header, auxiliary fields and the template fields (columns 7–9) and not preserved. For a fair comparison with other SAM compressors we removed auxiliary fields and set columns 7–9 as “*”, “0” and “0” respectively in all compression tests.

The results in Table 4 clearly indicate that the DNA sequence portion accounts for a minority of the disk space, yet is the primary purpose for the file. An obvious corollary to this is to consider whether we can lose or reduce the information content of the identifiers and quality values. Sequence identifiers have some initial use for detection of optical duplicates - the identifier typically encodes an X,Y location too - but beyond this it is used solely as a device for pairing sequences. The CRAM format solves this by removing names and keeping only the pairing information. Likewise the quality values may not need 40 separate levels for each base. CRAM provides multiple lossy methods. Some work has been done to evaluate the impact of using fewer quality levels [8].

Both Fqzcomp and Fastqz contain basic methods to lossily compress the quality strings, although Samcomp does not. The programs behave in slightly different manners. Fastqz rounds all quality above 1 to a multiple of Q where Q is the quantisation factor. This reduces the number of quality values and correspondingly increases the compression ratio. Fqzcomp has a similar approach, but instead every value above Q is encoded within Q of the original value, picking the value which happens to encode to the fewest bits given the current context. For example a quality value 37 with Q = 2 could be encoded as 35, 36, 37, 38 or 39. In practise this amounts to the same binning system as Fastqz unless the output is initially tuned on a few losslessly encoded quality strings.

## Methods

A FASTQ file consists of one or more sequences (typically millions) with each sequence being represented by an identifier, the DNA base calls, and the quality or confidence of each individual base call. A truncated example from the competition training set is shown below.

@SRR062634.2724179 HWI-EAS110_103327062∶6:13∶11133:13696/1 TGGAATCAGATGGAATCATCGAATGGACTGGAATGGAATCATTGAATGGACTCGAAAGG+GGGFGGFDGGGGGGFGFGGGGGGGGGGGGGGEFGGGGFGEDGGGFGGGFEDFGCDFDG? @SRR062634.2724180 HWI-EAS110_103327062∶6:13∶11133:11572/1 ATATAGTCCATTGTACTCCCTTGCTTAAATCTGGATCCCTGCAAATAAAAACATCTTCC+GGGGGGGGFGGGGEGGFGGGEGGFDGEAEGGEEEEBEEEEEEEEEEEEEEEEEEECCCC.

Each read is described in four lines of text. The first line, beginning with ‘@’, is an arbitrary text identifier. The format is machine specific, but it is typically a unique identifier constructed by joining run/flow-cell name and location (lane, X and Y coordinates) together. In this example, the NCBI “SRR” identifiers have also been prepended to the original sequence identifier. The second line holds the base call sequence consisting of A, C, G and T nucleotides with the occasional N. The third line consists of the ‘+’ character optionally followed by a copy of the sequence identifier (usually omitted). The fourth and final line holds the quality scores in Phred +33 format [26], i.e. a character with ASCII value 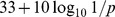, where 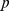 is the probability of error in the corresponding base and in the range 33 through 126 (‘!’through ‘∼’). Other phred encodings exist, such as Phred score +64, but are deprecated and not considered in this work. Practically speaking it is rare for Phred scores greater than 40 (ASCII ‘I’) to be utilized. In the competition data (from an Illumina instrument) all sequences are of the same length throughout the file, but this is not a requirement of the FASTQ format and some sequencing machines generate variable length records.

In common with previous work, the Fqzcomp and Fastqz programs both split FASTQ data into sequence identifiers, base-calls and quality scores, compressing the streams independently and in most cases in parallel. Each stream is passed through context models and into an arithmetic coder [27].

We define an 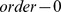 model to be one where probabilities are derived from the frequency of symbols with no context. An 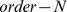 model counts the frequency of symbols given 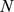 previous symbols. For example an order-0 model may assign 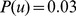 for the letter ‘u’ in English text, while an order-1 model may assign 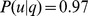, where ‘q’ is the context indicating that the letter ‘u’ is very frequent following the letter ‘q’.

The context models predict (assign probabilities to) consecutive symbols given the previous context and statistics collected within that context. If a symbol is assigned a probability 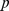, then the arithmetic coder chooses a code of amortized length 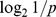 bits to represent it. This coding is provably optimal [28]. Thus, compression ratio depends on the quality of the predictions, 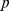, in turn depending on the accuracy of the context model.

### Fqzcomp

Fqzcomp uses a public domain byte-wise arithmetic coder from E. Shelwien (http://ctxmodel.net accessed on February 22, 2012). The context models are also derived from the same source, but have been heavily tuned to the type of data.

#### Identifiers

Fqzcomp compresses identifiers by using the previous identifier as a context to predict the current identifier. The identifier is tokenised into { *type*, *value*} pairs with *type* being one of alpha, numeric, leading zeros or punctuation (including spaces). For example: @ SRR 0 62634. 3364 HWI - EAS11 0 _ 103327062∶ 6 : 1∶ 1944 : 962/2.

The tokens are numbered 1 to N, producing N distinct models for the purposes of accumulating token specific statistics. When a token *type* is the same in the previous identifier the *value* is compared. Identical tokens are stored as type *match* while numerical values may be encoded as *delta* tokens when the current value is between 0 and 255 higher than the previous numeric value. Numeric values up to 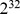 are encoded using four 8-bit models with larger numerical values being broken into 32-bit quantities to avoid overflow. *Alpha* and *punctuation* tokens are encoded a character at a time using a simple order-0 model; one which assumes each character is chosen independently and so requires no context.

In practise this does not work well for 454 data which mix a combination of letters and numbers together in order to achieve a base-64 encoding. This tends to produce a highly variable number of tokens. With hindsight an alphanumeric token type (matching [A-Za-z][A-Za-z0-9]* regular expression) would have worked better. An earlier identifier encoding method using simple string deltas is also available, performing better on 454 data.

#### Quality values

To encode quality values, let 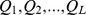 be the coded quality scores for a sequence of length 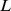.

For any specific score 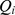 there is a strong correlation to the immediate previous few quality values 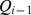, 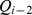 lessening the further back we go. We observed that many sequences ended in a run of score 2 (“#”), corresponding to a known issue with the Illumina base-caller [29].

All qualities are forced into the range 0 to 62, with 0 being exclusively required for bases called with “N”. Score 63 is used to represent a run of score 2 to the end of the sequence.

Most technologies have correlation between position and quality values, with quality typically reducing along the length of the sequence. It was also noted that sequences as a whole tend to be good or bad, so a sequence containing a number of low quality values is more likely to contain further low qualities even if the immediately previous values are high.

These correlations are combined to form successive levels of quality compression in Fqzcomp, selected by the user. Given 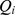 being the quality for the 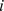
th base, the contexts used to predict 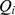 in Fqzcomp are:
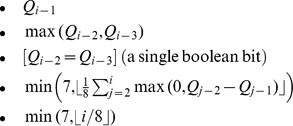



#### Sequence encoding

Fqzcomp encodes with an order-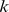 model, using the previous 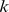 base calls to predict the next call. 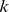 is a configurable parameter at run-time. Optionally the model can be updated with the reverse complement of the previous 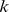 bases too. This gives a small improvement in compression ratio, but at a significant speed penalty. With high 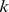 the program may learn the full genome, if sufficiently small. For example it is to be expected that on a 100 Mbp genome an order-14 model will be sparsely populated. Given sufficient depth the 14-mers in the model will represent the genomic sequence, allowing for accurate prediction of the next base so yielding high compression ratios. The same order-14 model on 3 Gbp genome however will have many random hits for each k-mer, only giving high accuracy within repeat regions and leading to poor compression. It is expected that most compression on large genomes is coming through common repeat sequences.

Fqzcomp also has the option to use an additional fixed size order-7 model, which is better for spotting short motifs and sequencing biases. There is no mixing step used here. Instead Fqzcomp encodes using the model containing the strongest probability bias (to any base type, not just the one being encoded). This is not very robust and is weak compared to context mixing, but achieves a small compression gain with a small CPU overhead.

The models used in Fqzcomp permit encoding of symbols 0, 1, 2 and 3 representing A, C, G and T). Each model uses 8-bit counters, meaning the combined model takes 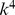 bytes. Base call N is simply encoded as 0 and fixed during decoding by observing that the associated quality value is 0 (this is enforced).

### Fastqz

Like Fqzcomp, Fastqz breaks the fastq file into three separate streams. However it contains an additional preprocessing step before the context modelling to reduce the data volume. This step is mainly a speed optimization and usually has a small cost in compression ratio.

The public domain libzpaq compression library was used for specifying the context models for the four streams in ZPAQ format (M. Mahoney, http://mattmahoney.net/dc/zpaq.html accessed on May 3, 2012). ZPAQ uses a context mixing algorithm based on PAQ [30] in which the bit-wise predictions of multiple independent context models are adaptively combined.

#### Identifiers

Differences between consecutive lines are encoded as a numeric field increment in the range 0-255, a match length, and trailing differences. For example, given the lines: @SRR062634.2724180 HWI-EAS110_103327062∶6:13∶11133:11572/1 @SRR062634.2724181 HWI-EAS110_103327062∶6:13∶11133:5630/1.

The second line is encoded as (18)(1)(51) ” 5630/1” (0), which means go to column 18, add 1 to the decimal string found there, then after this adjustment, copy the first 51 bytes, append the literal string, and terminate with a 0 byte (replacing the newline). In rare cases where an increment of more than 255 is required, then it is coded as a mismatch.

In fast mode, no further encoding is performed. In slow mode, the encoded names are modelled using a mix of four context models, each consisting of a hash of the column number, the current byte in the previous line, the previous bits in the current byte, and the last 1, 2, 3, or 4 bytes in the current line. The two low order models (1 and 2) map the context to a bit prediction and are updated by adjusting the prediction to reduce the error in inverse proportion to the context count. The two high order models are indirect context models. The context hash is mapped to an 8 bit state representing the bit history. The history is then mapped to an adaptively adjusted prediction. The outputs of the four models are combined by weighted averaging in the logistic domain, 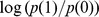. The weights are selected by an order 0 context that includes the column number, and adjusted on update to favour the better models.

#### Quality values

It was observed that the initial Q values tended to start with a common maximum value (38, ASCII “G”) and decline along the length of the sequence.

Scores are compressed by using byte codes to indicate runs of score 38 up to length 55, or groups of three scores in the range 35–38, or pairs of scores in the range 31–38, or single bytes for other scores, and finally a marker to indicate that the rest of the scores are 2 and are omitted. In fast mode, no further encoding is performed. In slow mode, the resulting codes are modelled using a mix of three direct context models as follows:

1. hash of 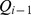, 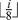

2. hash of 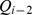, 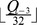

3. hash of 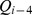, 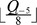

4. mixing weights: hash of 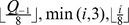

#### Sequence encoding

Fastqz starts by packing multiple base calls together, assigning A = 1, T = 2, C = 3 and G = 4. The only other code is N, which need not be coded because it always has a quality score of 0 and can be inserted during decoding. We pack either 3 or 4 bases together, whichever numerical packed value does not exceed 255. The coding is such that any sequence starting with G, CG, or CCG is coded in 3 bytes, but any other sequence is 4 bytes. The benefit of using 1234 over 0123 comes through self synchronization, so that overlapping reads that start at different locations will eventually be parsed into the same token sequence to allow compression. For example:

TGGA ATCA GAT GGA ATCA TCGA ATGG ACTG GAA TGGA ATCA

GGA ATCA GAT GGA ATCA TCGA ATGG ACTG GAA TGGA ATCA

GAAT CAGA TGGA ATCA TCGA ATGG ACTG GAA TGGA ATCA

AATC AGAT GGA ATCA TCGA ATGG ACTG GAA TGGA ATCA

In fast mode, no further encoding is performed. In slow mode, the encoded sequence is compressed using a mix of 6 models ranging from order 0 through order 5 bytes, or effectively order 4 through about 23 in bases. The order 0, 1, and 2 models are direct context models, using up to 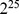 contexts, requiring 4 bytes of memory each. The order 3 model is an indirect context model, requiring 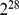 histories at one byte each. The order 4 model uses an indirect secondary symbol estimator. It adjusts the output of the previous model by mixing with the constant 1 in the logistic domain, where the pair of mixing weights is selected by the bit history in one of 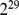 hashed contexts. The order 5 model uses a match model. A bit prediction is made by searching for the previous occurrence of the context in a 256 MB buffer and predicting the next bit with probability 1–1/(match length in bits). Contexts are looked up in a 256 MB hash table to find matches. Total memory usage is about 1.4 GB.

In both Fqzcomp and Fastqz the compression ratio of deep sequence data is strongly correlated with genome size, which can be compensated for by using additional memory. This is a problem which has partially been solved by [6].

#### Reference based encoding

The optimal compression of a set of sequence fragments involves a full identification of the relationships between all fragments; what their similarities are and whether they fit together to form some higher level structure (the source genome). This is largely the same problem solved by sequence assembly tools. One competitor, Daniel Jones, implemented his own sequence assembly algorithm (Quip) to permit data compression, but was largely hampered in the test data by low coverage and a relatively limited memory requirement.

If we have a known reference of the organism being sequenced we can instead implement a Lempel Ziv style compression algorithm [31]. This replaces portions of text with a coordinate and length into previously observed data, in this case the reference genome. In bioinformatics the equivalent are the sequence aligners or mappers, such as Smalt (http://www.sanger.ac.uk/resources/software/smalt/ accessed on August 29, 2012), BWA [32] or Bowtie [33].

Fastqz will optionally accept a reference genome of up to 4 GB with which it performs its own reference based mapping. If specified it must be present for both compression and decompression steps.

During compression, Fastqz will attempt to match sequences to the reference and encode them as a 32 bit pointer, a direction bit, and a list of up to four mismatched base positions. Matched bases are deleted from the sequences before compression and inserted after decompression.

To find matches, the reference is first packed 4 bases per byte using the code A = 0, C = 1, G = 2, T = 3 (deleting any N’s) and stored in up to 1 GB memory. The reference is divided into groups of 32 bases and a 1 GB hash table index is constructed, consisting of 256 M 27-bit pointers and 5 bit hash checksums as an optimization to detect 97% of collisions early. It searches 8 consecutive locations for an empty slot, resulting in about 6% of pointers being discarded when indexing a 2.9 Gb human genome in a 724 MB array.

To align, a rolling hash of the 100 byte reads is computed in both directions, swapping A with T and C with G in reverse. Starting at position 32, the hash is looked up and matches are ranked by the position of the fourth mismatch, breaking ties with the third, second, and first. The best ranked match is coded and the corresponding bases deleted from the read. If the fourth mismatch is less than the read length, then any remaining bases are coded as if not matching. If the fourth mismatch is less than half the read length, then the entire read is coded as if no match were found.

The list of alignments are coded in the format 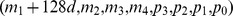 where 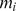 is the position of the 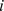
th mismatch in ascending order, or the read length +1 with less than 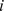 mismatches, 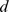 is 0 for a forward match and 1 for a reverse match, and 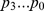 is the 4 byte pointer. An unmatched read is coded as a single 0 byte.

In fast mode, the encoded list of alignments is not compressed further. In slow mode, the list is compressed using a direct context model where the context is the parse state, the previous bits of the current byte, and the high 6 bits of the previous byte except when encoding the 2 low bytes of the pointer.

### Samcomp1

The Samcomp program takes a slightly different approach to Fastqz by offloading the issue of how to assemble or how to align to a third-party program; we used Bowtie2 for the competition but alternatives would work too.

The initial implementation (Samcomp v0.7) of this program requires a SAM or BAM file sorted by chromosome and position within the chromosome. Identifier and quality information is encoded as per Fqzcomp. For sequences it uses the SAM flags, position and CIGAR string to anchor each called base to a reference coordinate and encodes the base according to a per-coordinate model. As more and more data aligns to a specific reference coordinate the model improves in accuracy and the data compresses better. If a reference is known it is used to seed the initial model probabilities, otherwise they are seeded based on a low-order consensus context (for example simple GC content observations). Insertions and soft-clipped data use their own additional models.

This has some distinct advantages over simply encoding differences to a reference. Not requiring a reference avoids the necessity of storing the consensus sequence produced by a de-novo assembler. Given assemblies are typically deep, the model tunes well and the data compresses almost as well as supplying a reference, typically only taking up an extra 2 bits per consensus base. This is equivalent to shipping a compressed copy of the reference with the file. Additionally mapping to a closely related organism instead of the actual reference would generate many more differences. The per-position model will rapidly switch away from the claimed reference to the observed consensus instead, improving compression ratios. A similar gain can be seen when compressing data with systematic base-calling errors.

### Samcomp2

For the competition data set it was found that requiring position sorted data harmed compression of the sequence identifiers, so much so that the benefits of using a model per position were non-existent. Ideally the sequence identifiers would be omitted or replaced with lower complexity strings, but the SequenceSqueeze competition required a strictly lossless approach.

A second implementation of Samcomp (Samcomp2 v0.2) reads SAM files in any order, permitting the original name-ordered file to be used thus giving highly compressible sequence identifiers. To achieve this in limited memory the per-position model was removed and replaced by a simple reference difference model. The difference model uses a bit history of whether previous bases have been matching as context. Upon a mismatch the base-call itself is encoded, using the reference base as the context. This entry ultimately came top for lossless compression ratio on the SequenceSqueeze test data. However we feel it is not generally as useful as the original Samcomp program as Samcomp2 performs less well on positional sorted data and extremely badly when not supplied a reference.

## Discussion

We have demonstrated that there is a compression ratio vs speed and memory trade-off. For practical purposes saving the extra remaining few percent of compression is generally not worth it if you can get close to optimal compression in a fraction of the time. This is very apparent in the original SequenceSqueeze results when graphed on compression ratio against time (see Figure S1 in File S1).

The trade-off will depend on the use case. For long-distance data transfer such as uploading to the cloud, bandwidth is the limiting factor. Here we can compress, transfer and decompress in parallel with the total throughput being governed by the slowest of the three. The software presented here is ideally suitable for this task as the program usage is transitory so long term support is not an issue and additionally the requirement to immediately uncompress means it is easy to perform checksums to validate the transfer. The tools presented here have best-in-class tradeoffs between time and compression ratio such that Fqzcomp performs the best at low bandwidths and the fast mode of Fastqz performs best at high bandwidths.

The opposite use case is long time archival. The programs used need to be finalized and unchanging so the data can still be accessed years from now. One solution is to archive the programs along with the data, but more weight needs to be given to proven robust technology.

A final use case is for work-in-progress scenarios, requiring regular random access within a sequencing pipeline. Fqzcomp and Fastqz are only suitable for streaming, although adding random access is one obvious future improvement. However the bioinformatics community does not need yet more file formats. Ideally the existing and emerging formats (BAM, CRAM, cSRA) will incorporate ideas and methods presented here, while keeping their random access strength.

The rate of exponential growth in sequencing compared to disk technology and network bandwidth shows that any attempt to compress data is simply an interim measure. Compression buys us some time and it will always provide a cost saving, but ultimately the sequencing community will need to start prioritising data sets into those that are costly to produce and/or precious, and those that are cheap to reproduce. We envisage that in time many data sets will be viewed purely as a temporary transition between the raw DNA sample and final analysis, with file formats like VCF becoming a more mainstream end product and likely the topic of future compression research.

### Conclusion

We have shown methods for efficient compression of the three major components of FASTQ files and explained how these can be applied to reference based formats. While not the complete picture of SAM file compression, it is hoped that the techniques presented here may be used to improve existing SAM compressors.

We strongly believe that the creation of a public leader-board in the SequenceSqueeze competition had a direct and beneficial impact on the quality of all submissions. It was not uncommon for authors to leap-frog existing competitor’s entries, spurring them on to future improvements.

## Supporting Information

File S1 **Supplementary material.**
(PDF)
