# Supplementary material for: Compression of FASTQ and SAM Format Sequencing Data
Source: PLoS One. 2013 Mar 22;8(3):e59190. doi: 10.1371/journal.pone.0059190 (PMC3606433; doi:10.1371/journal.pone.0059190)
Supplement: File S1 — Supplementary material. (PDF) [file pone.0059190.s001.pdf]

SequenceSqueeze – Compression of FASTQ Format Sequencing Data

Supplementary material

Authors: James K. Bonfield and Matthew V. Mahoney

Data sets: stats

Name	Platform	Species	No. Seqs	Seq. Length	File size	Genome depth
SRR003177	454 GS FLX Titanium	Human	1,504,571	(Average) 564	1,754,042,560	0.28x
SRR007215_1	ABI SOLiD System 2.0	Human	4,711,141	25	689,319,444	0.04x
SRR027520_1	Illumina GA II	Human	24,246,685	76	5,055,253,238	0.61x
SRR065390_1	Illumina GA II	C.Elegans	33,808,546	100	8,819,496,191	33.8x
SRR013951_2	Illumina GA II	Human	18,212,437	76	3,663,611,628	0.46x
SRR062634_1	Illumina GA II	Human	24,148,993	100	6,345,444,769	0.80x

Table S1: data sets used for program comparison

SequenceSqueeze results

The evaluation machine used by the competition was an Amazon m2.xlarge instance with a separate 300GB mounted file-system for contest data and temporary storage. Amazon define this instance type as having 6.5 EC2 compute units (2 64-bit virtual cores of 3.25 ECU each), 17.1GB of memory with “moderate” I/O capacity. (*Source:* http://aws.amazon.com/ec2/instance-types/). A test linux system image was made available, identical to the judging image, for contestants to develop and test programs before submission.

The plots below have been generated from the table of results at www.sequencesqueeze.org. All entries from authors are shown, not just the best one. Entries that fail to uncompress without mismatch are omitted, except where an entrant had no programs that were 100% lossless – these were marked appropriately.

The cluster on the far left are the two reference based encoders – Fastqz and Samcomp. These include the time taken for the entire fastq → compress → decompress → fastq process, so this includes the bowtie2 alignment time. Fasqtz is demonstrably faster at performing alignments.

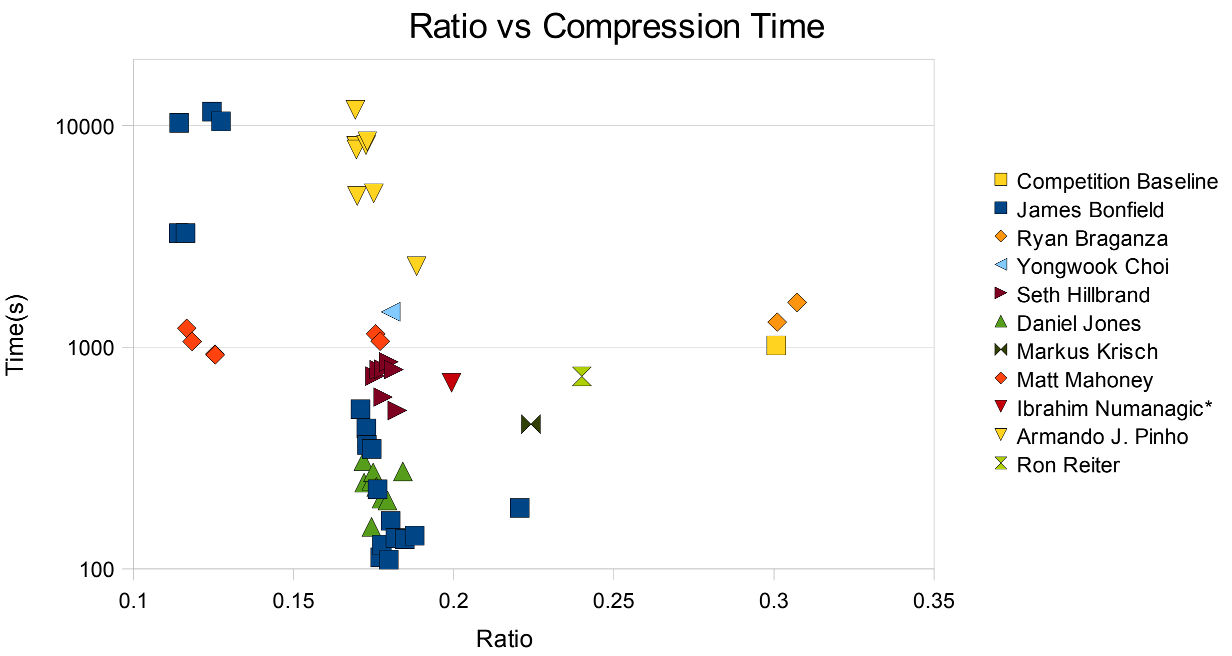

Figure S1

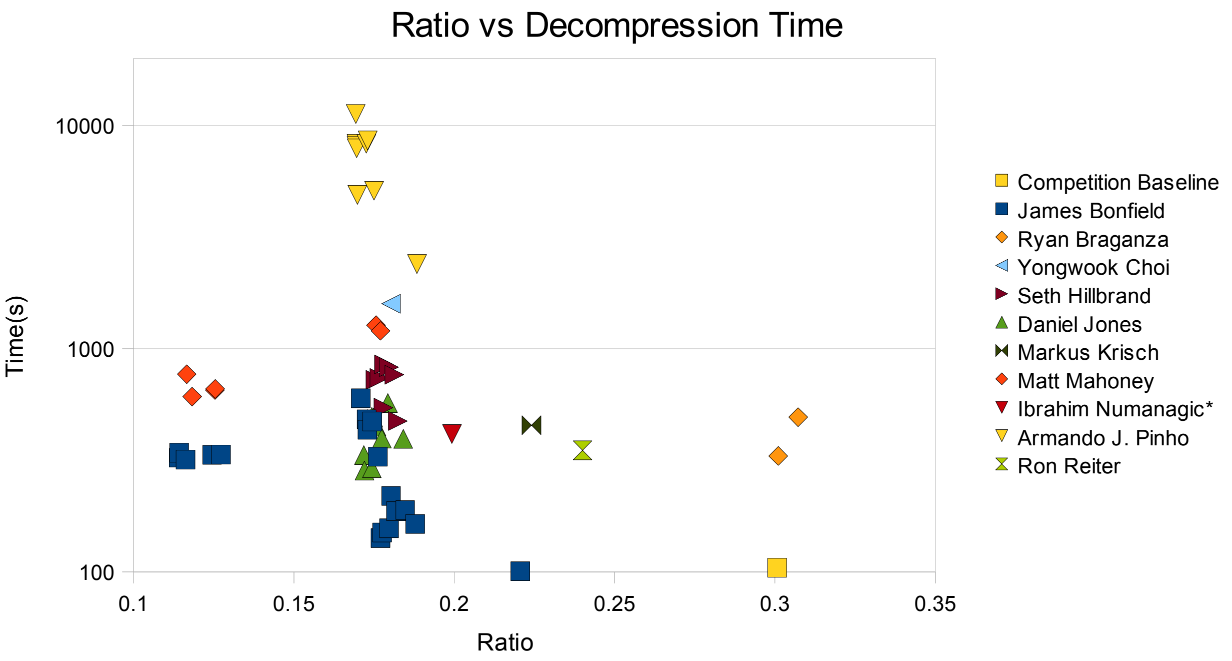

Figure S2

Despite many varied techniques, it is clear from compression times that there is a limit on compressibility, requiring exponentially more CPU to achieve only a linear and small improvement to ratio. Asymmetry of gzip (competition baseline) is clear. Most others are symmetric.

In the above plot fqzcomp appears to be the only program matching gzip on decompression speed. We believe this is likely due to both being I/O bound on the AWS test system. Our own tests show gzip to be faster at decompression.

Zooming up between ratio 0.17 and 0.19 more clearly shows the tradeoff between time vs ratio for the non-reference based compressors. From these the Pareto frontier consists of A.J. Pinho's IEETA entry, D. Jones' Quip program and J. Bonfield's fqzcomp. Programs may have been modified since the entry closed. (For example, Fqzcomp is 10-40% faster depending on options used.)

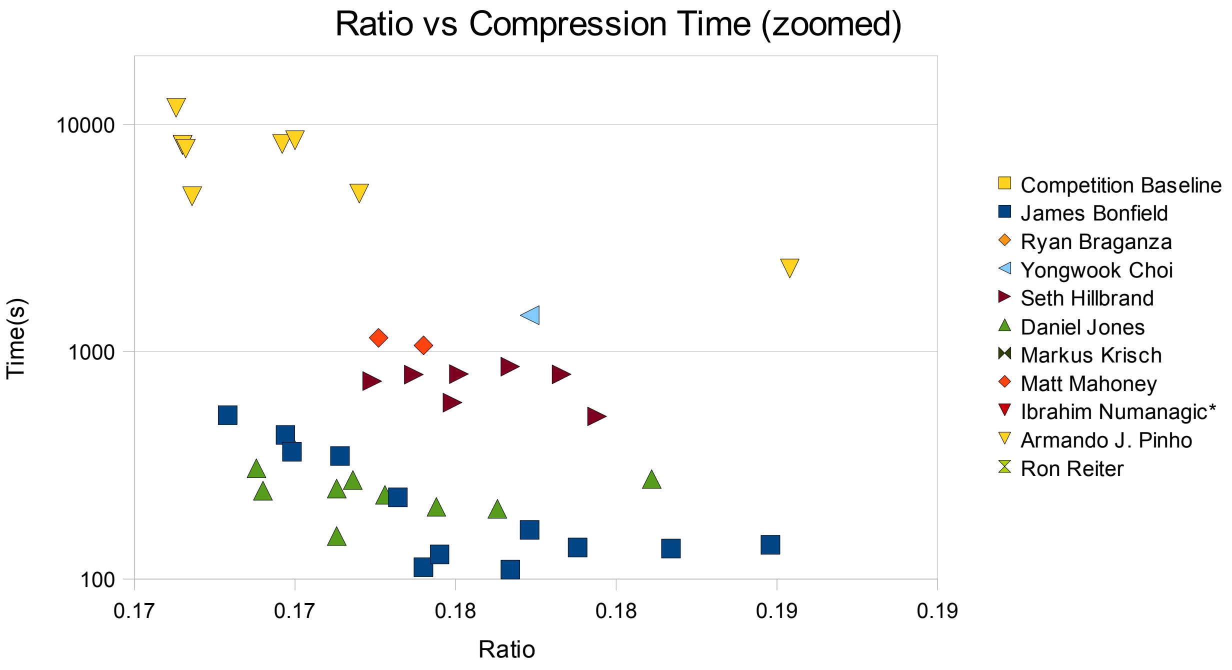

Figure S3

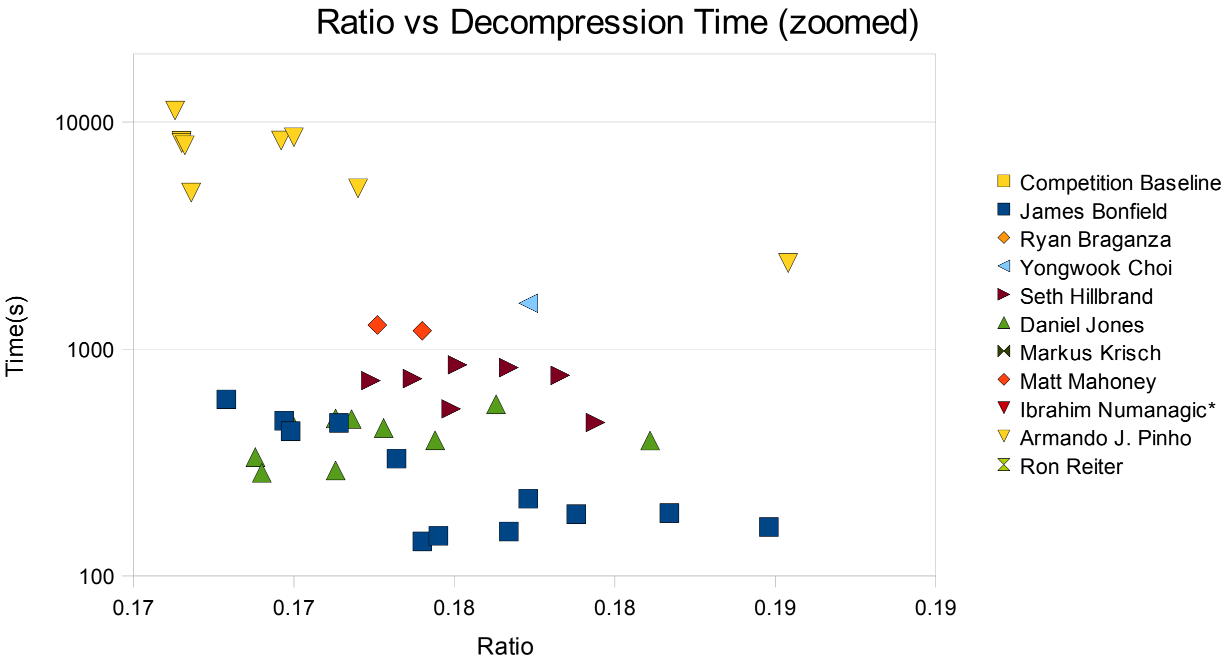

Figure S4

A similar picture is seen with compression ratio vs memory usage. Compression and decompression memory usage is largely symmetric, so we show only compression memory uage. Note that these memory figures are as quoted by the SequenceSqueeze web site, which erroneously listed them as the number of 1KB blocks; they are instead the number of 256-byte blocks.

Once again we see we rapidly reach a cliff, requiring exponential growth in memory for a linear decrease in size. The two reference based compression programs have the requirement of loading the reference genome into memory.

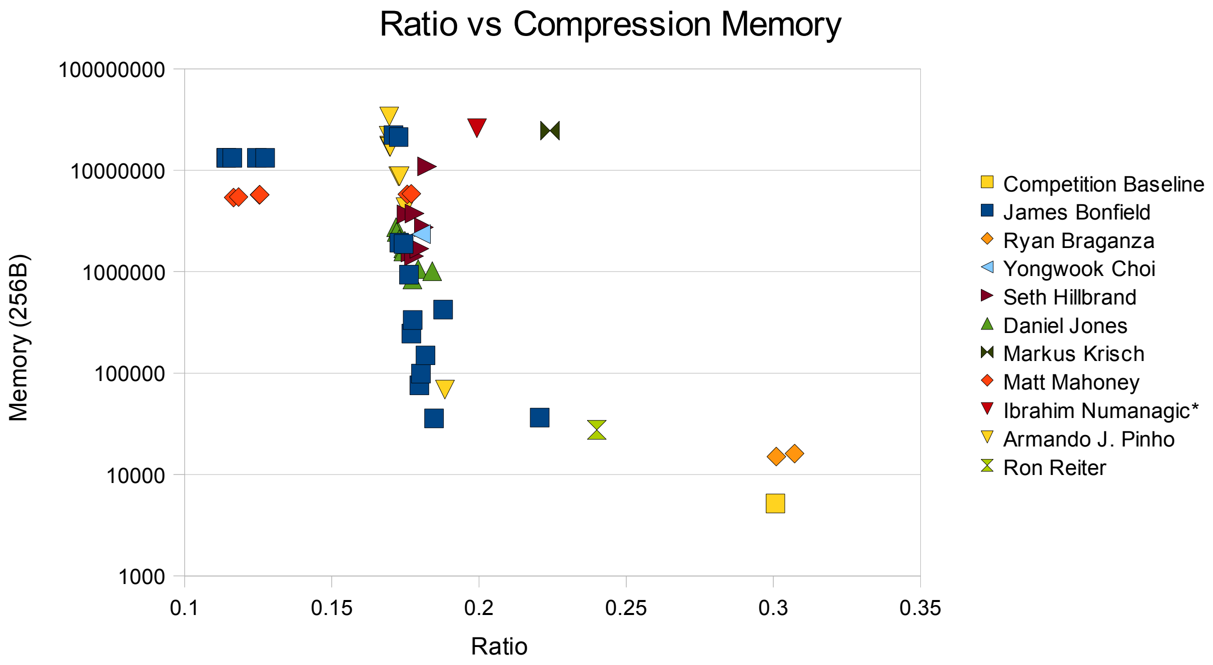

Figure S5

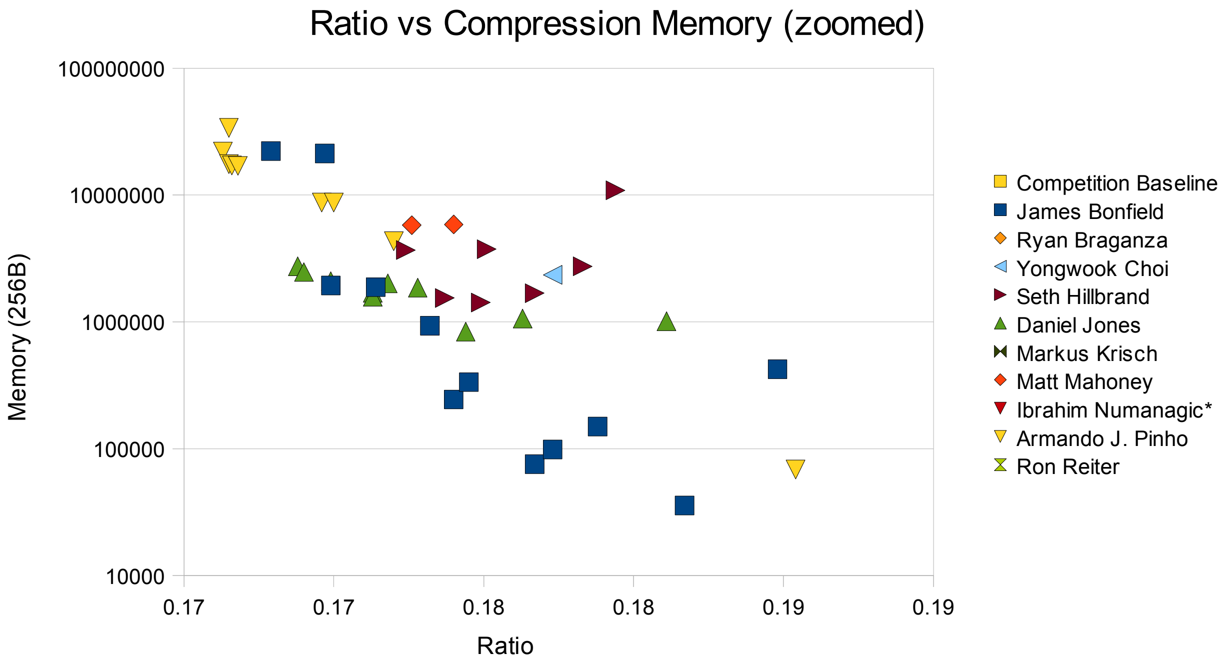

Figure S6

Bowtie2 alignment usage

Alignments for Samcomp and other SAM based aligners were produced using bowtie2 with the following script.

```
#!/bin/sh
bowtie=../prog/bowtie2-2.0.0-beta6/bowtie2
ref=../ce_indices/ce; # Edit as appropriate

mawk 'NR%4 == 1 {gsub(" ","|");gsub("/","~")} {print}' < $1 | \
$bowtie -x $ref -U - --sam-nohead --fast --threads 2 --mm > $1.sam
```

The purpose of the awk component is to combine a name consisting of both NCBI SRR identifier and original machine identifier into a single acceptable token. By default Bowtie only used the first word on the @identifier line and also removes the "/[12]" section. Replacing spaces and slashes before alignment permits reversal after encoding and decoding to allow lossless storage of identifiers.

Bowtie2 was considerably slower than the built-in aligner used by fastqz, but aligns more data.

Fastqz alignment benchmarks

The following results were obtained on the complete SRR062634 file (6,345,444,769 bytes). Fast mode decompression and unaligned compression are limited by disk I/O speed. CPU process times are shown in parenthesis. Fastqz tests were performed on a 2.0 GHz T3200 system with 3 GB under 32 bit Windows Vista.

**Fast**	**Unaligned**	**Fast**	**Aligned**
		183,313,663	alignments
639,049,273	base sequences	49,174,693	base sequences
251,697,610	headers	251,697,610	headers
867,178,255	quality	867,178,255	quality
1,757,925,138	total	1,351,364,221	total
Time 346s (117s CPU), 371s (111s CPU)		Time 1348s, 620s (150s CPU)	
**Slow**	**Unaligned**	**Slow**	**Aligned (submitted as ID 99)**
		105,063,319	alignments
503,239,070	base sequences	30,852,888	base sequences
47,861,283	headers	47,861,283	headers
574,112,937	quality	574,112,937	quality
1,125,213,290	total	757,890,427	total
Time 2357s, 2494s		Time 2231s, 1552s	

Table S2: FASTQZ v1.5 compression results for SRR062634_1.filt.fastq

The slow, aligned mode was submitted to the compression contest. The compression ratio was 0.1194 on this public file and 0.1166 on the withheld contest data. Run time was reported as 1218 seconds to compress and 769 seconds to decompress. Memory usage is reported as 5398224, apparently in units of 256 bytes. FASTQZ uses about 1.5 GB memory.

Producing the alignment adds significant time to the preprocessing stages. However in full slow compression mode this reduces the overall time spent due to the data volume presenting to the ZPAQ stage being smaller.

Fqzcomp parameter space

Fqzcomp has separate parameters controlling the compression level for sequence names (identifiers), base-calls and quality values. Additionally for base-call compression it may use a single or double stranded model and it may optionally encode using a single model or with a pair of low + high order models. This gives a considerable search space to explore.

To choose appropriate low, mid and high compression ratio parameters we produced charts with consistent name ("n") and quality ("q") parameters, along with consistent choices of single vs double ("b") strand and single or paired ("+") model, but varied the sequence ("s") order to chart lines of compression ratio vs time. We tested this using two Illumina data sets (shallow and deep) and a 454 data set.

"s*" refers to **-s1** to **-s8** parameters except on slower compression modes where **-s6** to **-s8** was used (visible in the lines that contain just 3 data points). The model used for predicting base-calls is order 7 + *x* where *x* is the value after **-s.** E.g. **-s1** uses an order-8 model and **-s8** uses an order-15 model.

"+" refers to **-s1+** to **-s8+** parameters, indicates the use of an additional shorter order-7 model. No context mixing is used. Instead the program encodes using either the order-7 model or the order-8 to order-15 model (as indicated by the -s*num*), depending on which appears to have the most extreme probability bias (for any base type, not just the one being encoded).

"b" refers to the **-b** parameter, specifying that updates to the sequence model should take place on both strands.

"q1", "q2" and "q3" refer to the **-q1**, **-q2** and **-q3** parameters.
"q1" uses the first 2 quality contexts described in the paper (section 2.1.2)
"q2" uses the first 4 quality contexts.
"q3" uses all 5 quality contexts.

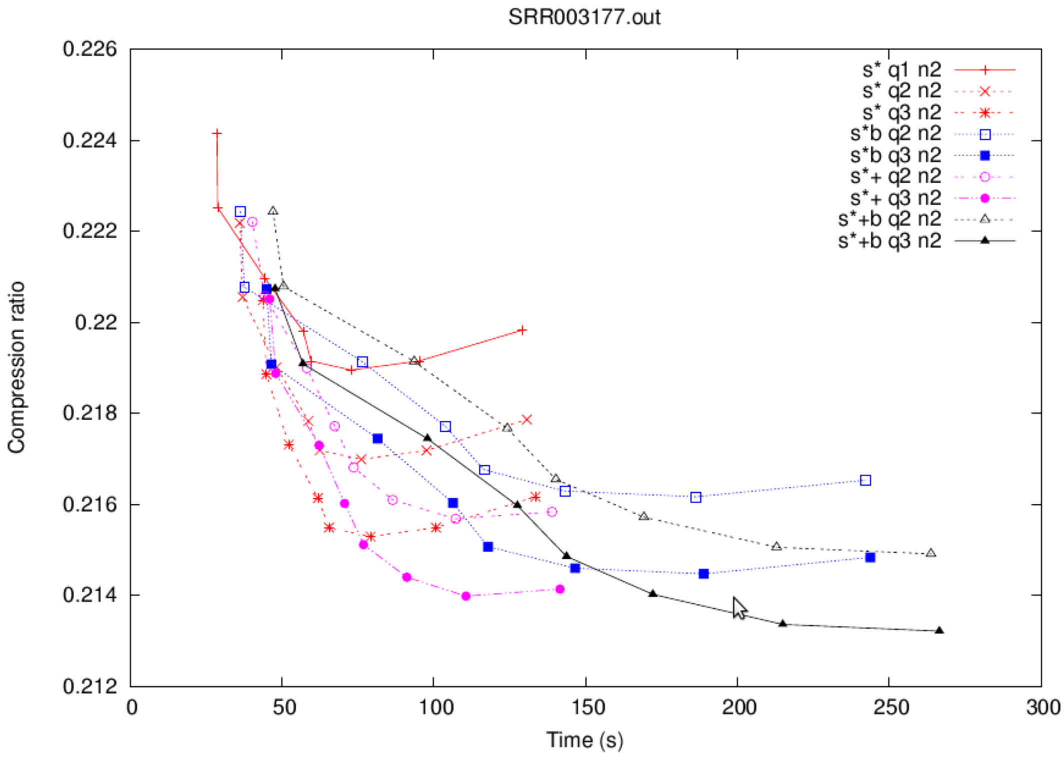

Figure S7: Roche 454 data

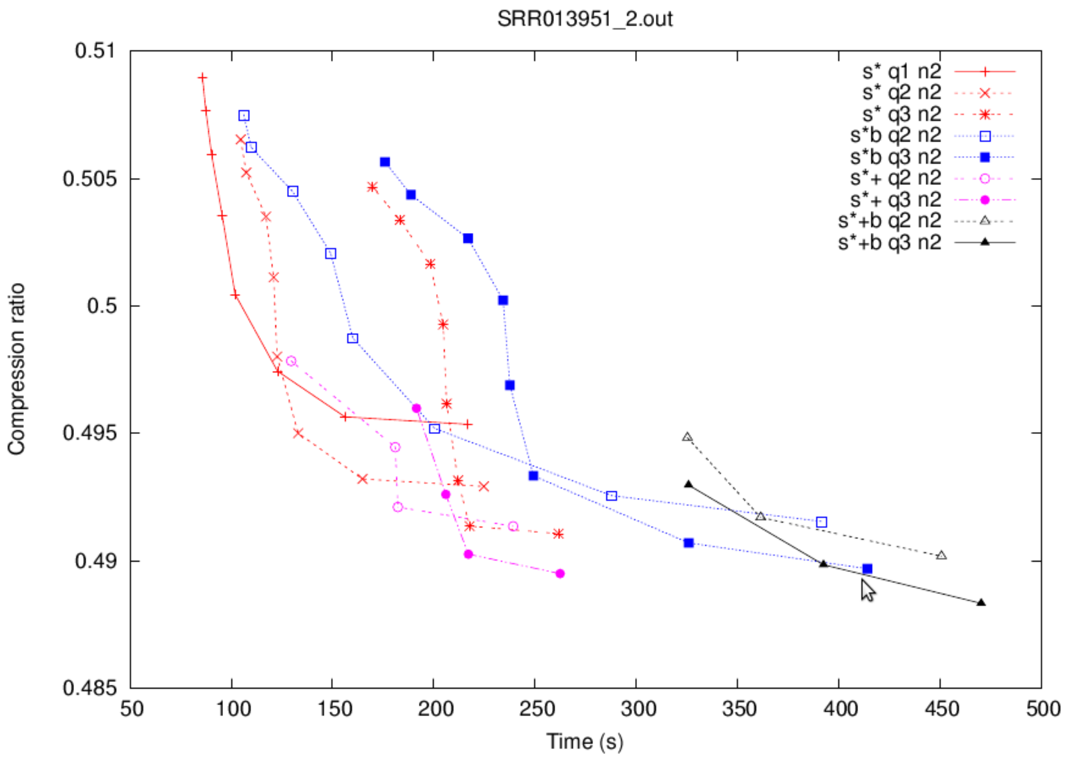

Figure S8: Illumina human data (low coverage)

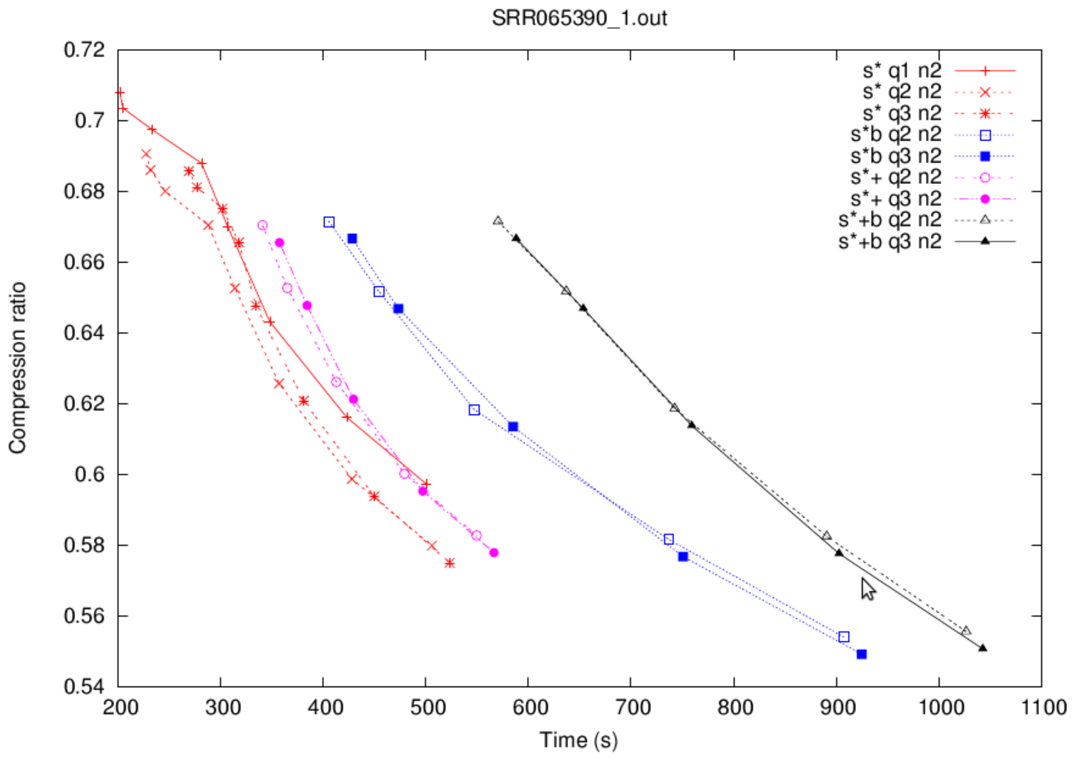

Figure S9: Illumina C.Elegans data (high coverage)
